# Acute Spontaneous Spinal Epidural Hematoma in a Patient on a Direct Oral Anticoagulant (DOAC): A Case Report

**DOI:** 10.7759/cureus.88105

**Published:** 2025-07-16

**Authors:** Zubair Ahmad, Junaid Ahmad, Awais Aslam, Shoukat Ullah, Uzair Khan, Khyle Cole, Muhammad Yasir Rafiq, Shahid Kausar

**Affiliations:** 1 Department of Stroke Medicine, Dudley Group NHS Foundation Trust (DGFT), Dudley, GBR; 2 Department of Internal Medicine, Hayatabad Medical Complex Peshawar, Peshawar, PAK; 3 Department of Emergency Medicine, Dudley Group NHS Foundation Trust (DGFT), Dudley, GBR; 4 Department of Neurology, Dudley Group NHS Foundation Trust (DGFT), Dudley, GBR; 5 Department of Internal Medicine, Bacha Khan Medical Complex, Swabi, PAK; 6 Department of Internal Medicine, Khyber Institute of Medical Sciences, Kohat, PAK; 7 Department of Radiology, Dudley Group NHS Foundation Trust (DGFT), Dudley, GBR; 8 Department of Stroke Medicine, Russells Hall Hospital, Dudley Group NHS Foundation Trust (DGFT), Dudley, GBR

**Keywords:** anticoagulant, atrial fibrillation, cervical spine, magnetic resonance imaging, mri diagnosis, spinal epidural hematoma, stroke

## Abstract

Acute spontaneous spinal epidural hematoma (SSEH) is a rare but serious condition that can mimic stroke or carotid artery dissection (CAD), potentially leading to misdiagnosis and inappropriate therapy. We report the case of a 75-year-old male with atrial fibrillation on Edoxaban who awoke with neck pain and, approximately 80 minutes later, developed sudden right-sided weakness. Neurological examination showed right upper limb (RUL) weakness (3/5 proximally, 0/5 distally), right lower limb (RLL) paralysis (0/5), hypotonia, but intact sensation, with a National Institutes of Health Stroke Scale (NIHSS) score of 6 - findings that supported conservative management in the absence of progressive deterioration. Initial CT head and CT angiogram were unremarkable, ruling out intracranial hemorrhage and carotid dissection. Aortic dissection was also excluded by CT aortogram. Although uncontrolled hypertension or other acute risk factors were not identified (blood pressure (BP) 157/81 mmHg), chronic anticoagulation likely contributed to SSEH. Cervical spine magnetic resonance imaging (MRI) performed on day 6 revealed a C3-C5 epidural hematoma compressing the cord. Neurosurgical consultation recommended conservative management due to improving strength and absence of worsening symptoms. Follow-up MRI at six weeks confirmed complete hematoma resolution with residual mild myelopathy. At discharge, after a 10-day hospital stay, the patient’s neurological function had improved to 4/5 strength in the RUL and 3/5 in the RLL, and he was transferred for rehabilitation. This case underscores the importance of considering SSEH in anticoagulated patients with acute neck pain and hemiparesis, even when initial imaging for stroke or CAD is negative. Timely MRI and a multidisciplinary approach are crucial to avoid misdiagnosis and optimize outcomes.

## Introduction

Acute spontaneous spinal epidural hematoma (SSEH) is a rare but potentially devastating neurological emergency, accounting for less than 1% of all space-occupying spinal cord lesions [1]. It arises from bleeding into the epidural space, which compresses the spinal cord and leads to acute neurological deficits. The most widely accepted mechanism involves rupture of the fragile epidural venous plexus due to a sudden increase in venous pressure, although arterial sources have also been implicated [2,3]. Administration of thrombolytic agents in such cases can exacerbate bleeding and hematoma expansion, worsening neurological outcomes, and therefore recognizing SSEH as a stroke mimic is clinically critical [4].

SSEH often presents with acute neck or back pain, rapidly progressing to motor and sensory deficits. Hemiparesis is an uncommon presentation and may mimic ischemic stroke or carotid artery dissection [5]. Early differentiation from these more common vascular events is crucial to avoid harmful interventions like thrombolysis. On clinical grounds, the presence of severe neck or interscapular pain at onset, coupled with unilateral weakness without facial involvement, may suggest a spinal rather than cerebral etiology [6]. However, these distinguishing features are subtle and often overlooked in the emergency setting.

Imaging plays a central role in diagnosis. While non-contrast head CT and CT angiography are performed initially to rule out stroke or carotid dissection, these are typically unremarkable in SSEH. Magnetic resonance imaging (MRI) of the cervical and thoracic spine is the gold standard, offering superior soft tissue resolution to identify the hematoma and assess cord compression [7,8]. Therefore, early consideration of MRI in suspected stroke mimics is recommended.

Anticoagulation therapy is a well-recognized risk factor for SSEH, which has an annual incidence of about 0.1 per 100,000 and accounts for less than 1% of spinal epidural space-occupying lesions, though 40%-50% of cases remain idiopathic [1,2]. Direct oral anticoagulants (DOACs) such as rivaroxaban and apixaban have been increasingly implicated, with patients typically presenting with acute neck or back pain followed by progressive neurological deficits, and elevated INR on admission correlating with worse outcomes. In such cases, early recognition, prompt reversal of anticoagulation with agents like prothrombin complex concentrate or andexanet alfa, and timely surgical intervention - ideally within 12-36 hours - are critical to optimizing neurological recovery [1,9]. Awareness of this association is vital in evaluating anticoagulated patients presenting with sudden-onset neurological deficits and neck pain.

Given the risk of irreversible neurological injury and the potential for misdiagnosis leading to inappropriate treatment, SSEH should be included in the differential diagnosis of acute hemiparesis, particularly in anticoagulated patients. Early recognition, prompt imaging, and appropriate management can significantly improve outcomes.

## Case presentation

A 75-year-old male with a past medical history of ischemic heart disease (IHD) with percutaneous coronary intervention (PCI) in July 2024, chronic obstructive pulmonary disease (COPD), carotid endarterectomy, essential hypertension, atrial fibrillation, and a transient ischemic attack (TIA), was brought to the emergency department (ED) by ambulance after waking up at 6:00 AM with lower head and neck pain, radiating to the shoulder. At approximately 7:20 AM, the patient developed weakness in his right arm and leg. He was on Edoxaban for non-valvular atrial fibrillation. There was no history of trauma or fall. A FAST-positive assessment was noted, and the stroke team was alerted by the ED team for immediate evaluation.

Upon examination at 7:44 AM, the patient was fully conscious (Glasgow coma scale 15/15), with stable hemodynamics. He was found to have equal and reactive pupils, with right upper limb (RUL) power of 3/5 proximally and 0/5 distally, and right lower limb (RLL) power of 0/5, with hypotonia on the right side. There was no upper motor neuron (UMN) facial palsy or inattention, and sensation was intact. The patient’s cerebellar examination appeared normal in the right upper limb (RUL), but could not be assessed in the RLL due to weakness. The National Institutes of Health Stroke Scale (NIHSS) score was 6. The last dose of Edoxaban had been taken more than 24 hours earlier. Initial assessment suggested a stroke within the window period for thrombolysis and thrombectomy, or potentially a carotid dissection. A CT head was requested to rule out a bleed, and a CT angiogram was ordered to assess for large vessel occlusion (LVO) or carotid dissection. The CT head and CT angiogram were normal, and no LVO or carotid dissection was identified.

Following discussion with the stroke consultant, it was decided that the patient was not a candidate for thrombolysis given the last dose of Edoxaban in 24-48 hours, and the differential diagnosis included a possible aortic dissection. A CT aortogram was urgently performed, which returned normal. The patient was transferred to the stroke ward for further management.

On clinical review in the stroke ward at 11:58 AM, vital signs were stable with respiratory rate 19, oxygen saturation 98% on room air, blood pressure 157/81 mmHg, pulse 88, and temperature 36.6 °C. Neurological examination revealed no inattention, normal speech, and no facial droop; however, RUL power was 3/5, and RLL power was 0/5. Sensation remained intact, with tense right sternocleidomastoid muscles. The diagnosis of a left lacunar stroke (LACS) and torticollis was made, and the patient was started on aspirin 300 mg. An urgent MRI head was requested.

**Figure 1:**
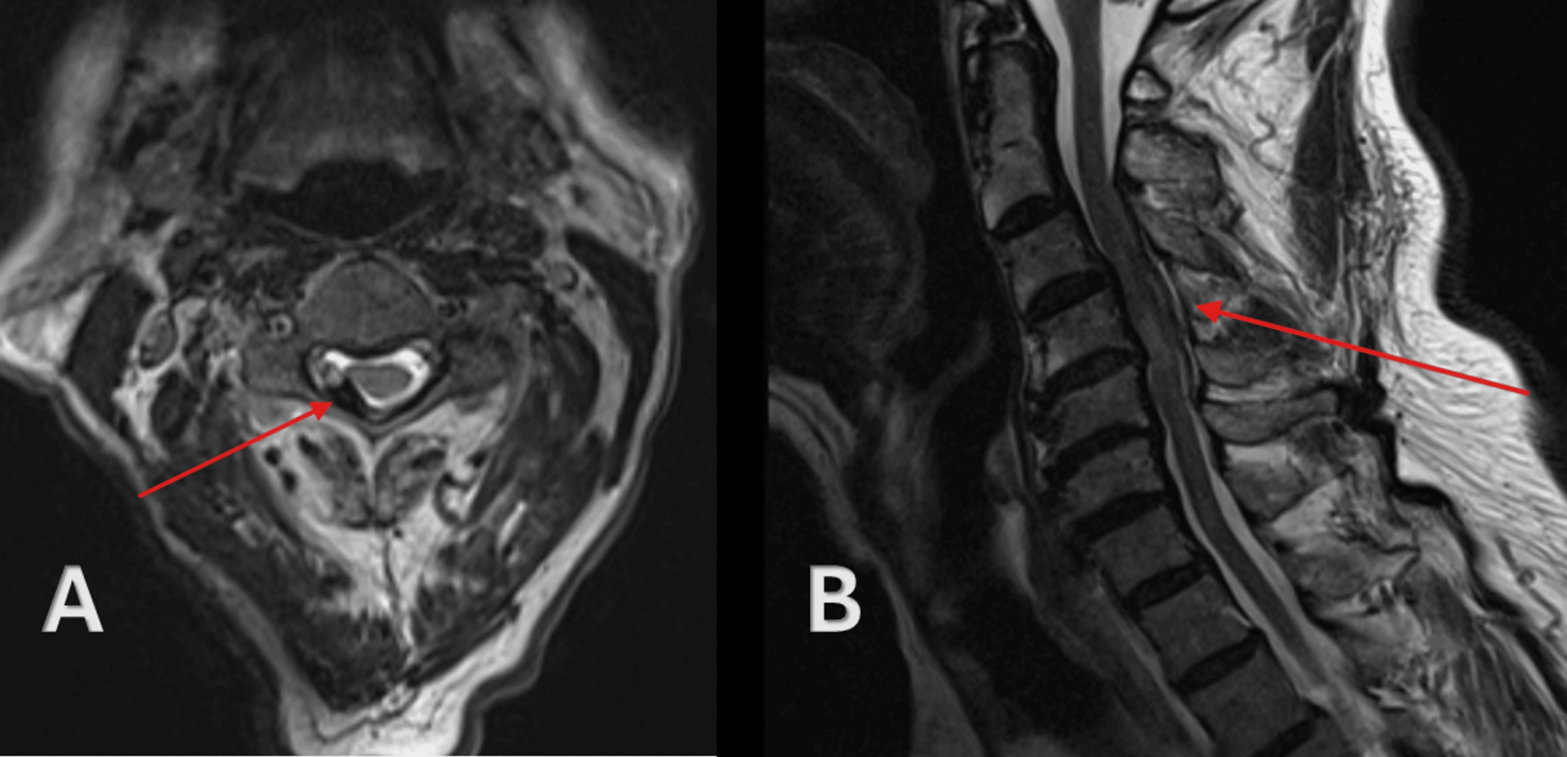
Axial T2-weighted MRI of the cervical spine (A) showing a spinal epidural hematoma, and sagittal T2-weighted MRI (B) demonstrating cervical spinal cord compression with associated myelopathic signal changes. MRI, magnetic resonance imaging

The following day, the MRI head was normal, and a diagnosis of stroke was ruled out. Endoxaban was restarted, but the patient continued to experience weakness on the right side and persistent shoulder pain. An urgent MRI of the cervical spine was performed approximately 6 days after symptom onset, which reported mid-cervical spondylitic changes resulting in cord compression and associated myelopathic changes (Figure 1). These spondylitic changes were initially thought to explain the patient’s symptoms; however, after neurosurgical MDT review, the findings were re-interpreted as an epidural hematoma causing the compression. Urgent spinal surgical review was advised.

A neurosurgical referral was made, and the case was reviewed in a multidisciplinary team (MDT) meeting, where it was determined that a C3-C5 epidural hematoma - rather than spondylitic changes - was responsible for the spinal cord compression. Edoxaban was stopped, and conservative management with close neurological monitoring was chosen given the patient’s clinical stability, gradual improvement in motor strength, and high surgical risk due to comorbidities. The patient remained stable throughout hospitalization without any worsening of neurological deficits.

Over the following days, progressive improvement in right-sided weakness was observed, with the RUL improving to 3/5 and the RLL to 2/5. On neurosurgical advice, a contrast-enhanced MRI of the cervical spine was performed, which showed interval reduction in the hematoma volume at C3-C5 and resolving myelopathy, with no evidence of an underlying vascular malformation. Conservative management was continued.

At discharge, after an 11-day hospital stay, the patient demonstrated significant neurological recovery, with RUL power of 4/5 and RLL power of 3/5 on the Medical Research Council (MRC) scale, and was able to perform independent transfers. He was transferred to a rehabilitation center for continued recovery. Rehabilitation goals and expected prognosis were discussed with the patient and his family, highlighting the importance of ongoing physiotherapy and the anticipated gradual improvement in function.

Outcomes and follow-up

**Figure 2:**
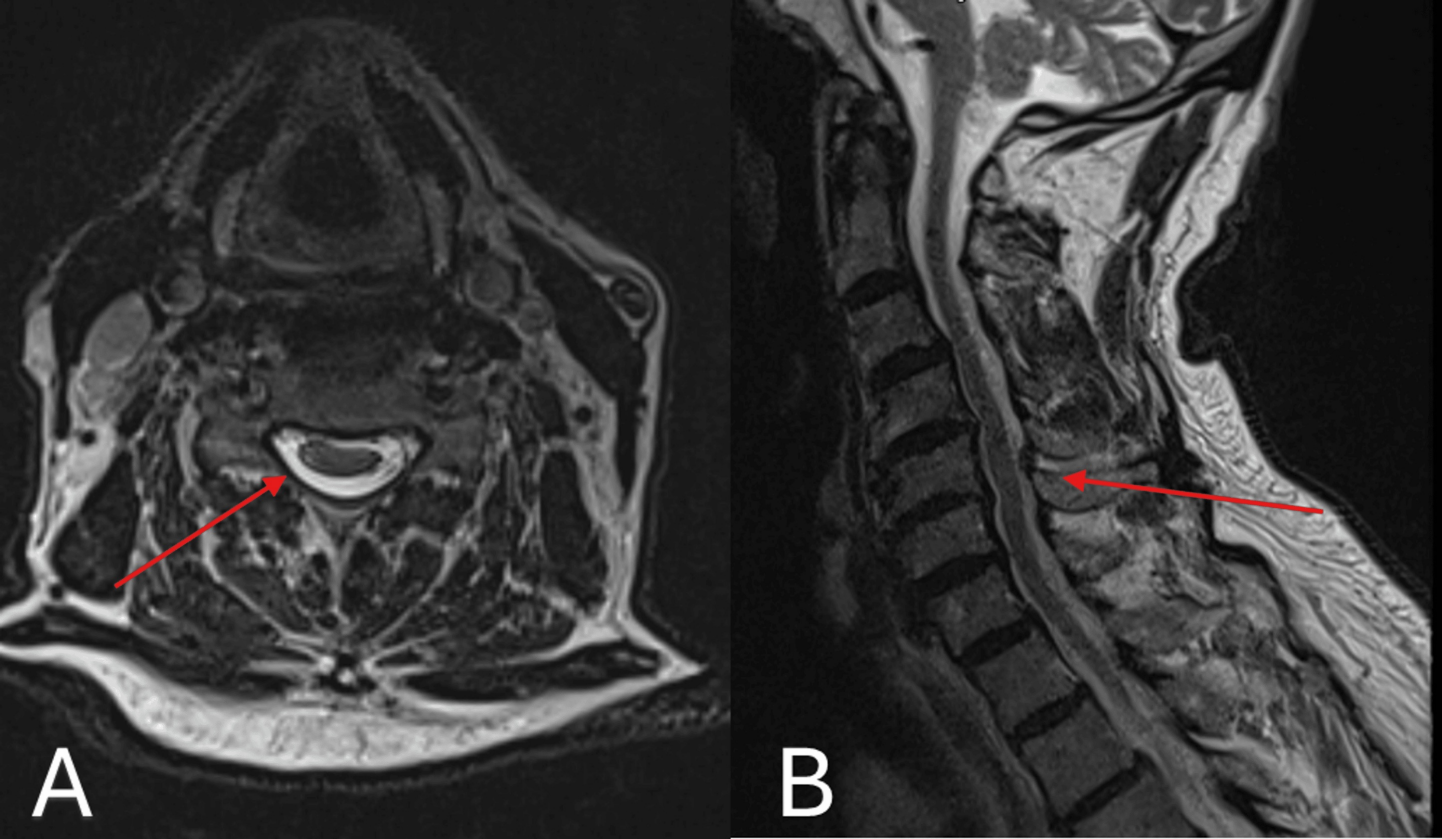
Axial T2-weighted MRI (A) showing resolution of the spinal epidural haematoma, and sagittal T2-weighted MRI (B) demonstrating residual, though reduced, myelopathic signal changes at the C4-C5 level. MRI, magnetic resonance imaging

A follow-up MRI was performed six weeks after the initial presentation to evaluate the feasibility of resuming anticoagulation therapy. The imaging demonstrated complete resolution of the spinal epidural hematoma, with persistent but reduced myelopathic signal changes at the C4-C5 level (Figure 2). The case was subsequently reviewed in an MDT meeting, and anticoagulation therapy was reinstated.

## Discussion

SSEH most commonly involves the C6 and T12 levels of the spine, likely reflecting regions of increased mechanical stress and vascular vulnerability [10,11]. However, as seen in this patient’s C3-C5 lesion, SSEH can occur at atypical levels and still produce significant cord compression and neurological deficits. While SSEH typically presents with paraparesis or quadriparesis, this case presented with hemiparesis, which, although rare, has been documented in the literature and is thought to result from unilateral cord compression in the cervical region [12].

The etiology of SSEH may involve rupture of the epidural venous plexus due to increased intrathoracic or intra-abdominal pressure, though coagulopathy, vascular malformations, and anticoagulant medications, as in this case, are recognized risk factors [1]. The differential diagnosis includes ischemic stroke, carotid or aortic dissection, spinal disc disease, infection, and neoplasm [2,13]. Misdiagnosis of SSEH as an acute ischemic stroke has been reported in up to 50% of cases presenting with hemiparesis, and inappropriate thrombolysis has been shown to worsen hematoma expansion and outcomes [9].

On imaging, SSEH typically appears on MRI as a hyperintense dorsal or ventral extradural collection compressing the cord, often without parenchymal infarction, while ischemic stroke is characterized by focal parenchymal diffusion restriction without epidural signal abnormalities [2,9,13,14]. Prompt MRI within 12-48 hours of symptom onset is crucial to minimize irreversible neurological damage [15].

Management of SSEH depends on neurological status and progression. Surgical decompression is indicated in patients with severe or worsening deficits, large hematomas, or unstable neurological findings, while conservative management may be appropriate in stable patients with improving symptoms and high surgical risk, as in this case [16-18]. Anticoagulants and antiplatelets should be discontinued immediately, and neurosurgical evaluation is essential for individualized decision-making.

## Conclusions

This case highlights that SSEH should be considered in anticoagulated patients presenting with acute neck pain and unilateral weakness, especially when initial stroke imaging is inconclusive or normal. In our patient, conservative management was chosen based on neurological stability, gradual improvement, and elevated surgical risk, emphasizing the need for individualized, patient-centered treatment decisions. The favorable outcome underscores the vital role of multidisciplinary collaboration among neurology, radiology, and neurosurgery teams in managing such complex cases. Given the risk of irreversible deficits with delayed diagnosis, early cervical spine MRI remains critical to avoid mismanagement, including inappropriate thrombolysis, and facilitate timely intervention. This case also supports the need for greater awareness and potential incorporation of SSEH into diagnostic pathways and clinical guidelines for anticoagulated patients presenting with stroke-like symptoms.
